# A potential panel of six-long non-coding RNA signature to improve survival prediction of diffuse large-B-cell lymphoma

**DOI:** 10.1038/srep27842

**Published:** 2016-06-13

**Authors:** Jie Sun, Liang Cheng, Hongbo Shi, Zhaoyue Zhang, Hengqiang Zhao, Zhenzhen Wang, Meng Zhou

**Affiliations:** 1 College of Bioinformatics Science and Technology, Harbin Medical University, Harbin 150081, PR China

## Abstract

Long non-coding RNAs (lncRNAs) represent an emerging layer of cancer biology and have been implicated in the development and progression of cancers. However, the prognostic significance of lncRNAs in diffuse large-B-cell lymphoma (DLBCL) remains unclear and needs to be systematically investigated. In this study, we obtained and analyzed lncRNA expression profiles in three cohorts of 1043 DLBCL patients by repurposing the publicly available microarray datasets from the Gene Expression Omnibus (GEO) database. In the discovery series of 207 patients, we identified a set of six lncRNAs that was significantly associated with patients’ overall survival (OS) using univariate Cox regression analysis. The six prognostic lncRNAs were combined to form an expression-based six-lncRNA signature which classified patients of the discovery series into the high-risk group and low-risk group with significantly different survival outcome (HR = 2.31, 95% CI = 1.8 to 2.965, p < 0.001). The six-lncRNA signature was further confirmed in the internal testing series and two additional independent datasets with different array platform. Moreover, the prognostic value of the six-lncRNA signature is independent of conventional clinical factors. Functional analysis suggested that six-lncRNA signature may be involved with DLBCL through exerting their regulatory roles in known cancer-related pathways, immune system and signaling molecules interaction.

Diffuse large B-cell lymphoma (DLBCL) is the most common and aggressive subtype of non-Hodgkin lymphoma (NHL), constituting 30–58% of all diagnosed NHL cases^1^. The current standard treatment of DLBCL is a combination of Rituximab with traditional chemotherapy of cyclophosphamide-doxorubicin-vincristine-prednisone (R-CHOP). Although R-CHOP has been proven to be an effective treatment for this disease, nearly 40% of DLBCL patients still faced the failure of standard therapy and ultimately died from their disease^2^. Therefore, it is important to make risk stratification for DLBCL patients to identify high-risk patients who are unlikely to be cured with standard therapy and would therefore benefit from more effective therapy.

Recent advances in genomic and transcriptomic analysis have accelerated the discovery and identification of various types of non-coding RNAs (ncRNAs). Long non-coding RNAs, a recently discovered major class of ncRNAs, were arbitrarily defined as RNA transcripts of more than 200 bp in length that lack or have little protein coding capacity^3^. Growing evidence indicated that lncRNAs can function as a critical player of genome regulatory network to participate in the process of gene regulation, post-transcriptional regulation and epigenetic regulation^4^,^5^. Dysregulated lncRNAs expression have been observed frequently in tumors when compared to normal adjacent tissues, implying their oncogenic and tumor suppressor roles in cancer development^6^,^7^. Recently, transcriptome sequencing analysis has revealed the aberrant expression of lncRNAs between DLBCL and normal B cells^8^. Another lncRNA, *PEG10*, was reported to be unregulated in DLBCL compared with normal tissues^9^, implying their perspective in diagnostics and prognosis as potential biomarkers in DLBCL. However, the prognostic significance of lncRNAs in DLBCL remains unclear and needs to be systematically investigated.

In order to study the prognostic significance of lncRNAs for risk stratification of DLBCL, we obtained and analyzed lncRNA expression profiles on a large number of DLBCL patients by repurposing the publicly available microarray datasets from the Gene Expression Omnibus (GEO) database. Our analysis identified six prognostic lncRNAs that were significantly associated with survival outcome of DLBCL patients from the discovery series by using Cox regression analysis. We then developed a six-lncRNA signature by the risk score model method based on the expression levels of these six prognostic lncRNAs which could distinguish patients with good and poor survival. Moreover, additional results using the internal testing series and two independent non-overlapped patient datasets further confirmed the prognostic value of the six-lncRNA signature.

## Results

### Initial prognostic lncRNAs screening using lncRNA expression profiles and survival data in the discovery series

**Figure 1:**
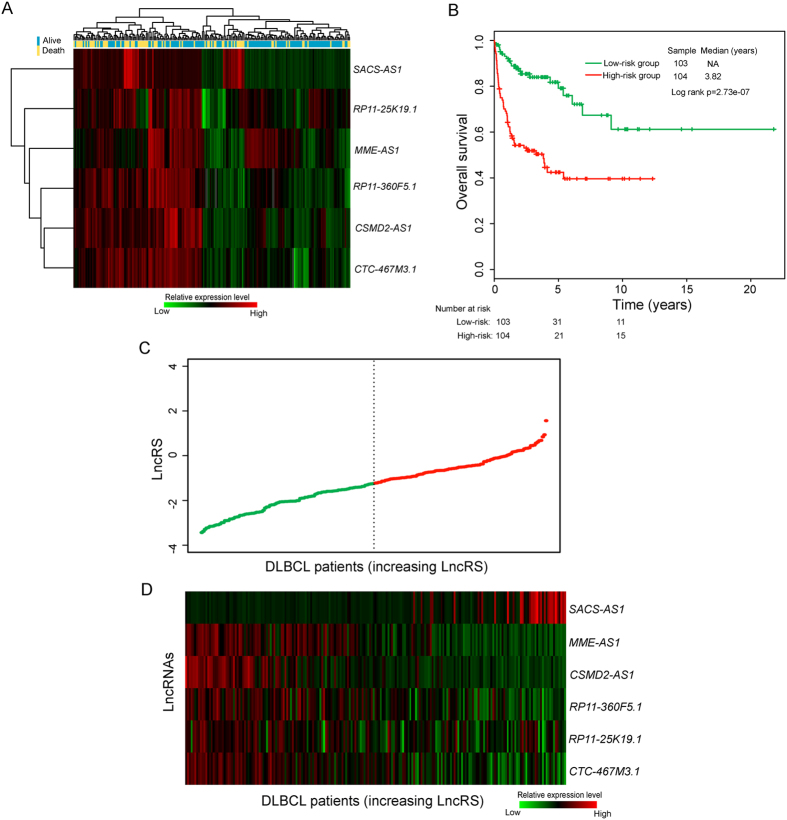
Prognostic evaluation of the six-lncRNA signature in the discovery series. (**A**) Hierarchical clustering analysis for 207 DLBCL patients based on expression levels of six prognostic lncRNAs in the discovery series. (**B**) Kaplan–Meier survival curves for patients in the discovery series. (**C**) The distribution of LncRS. (**D**) The expression heatmap of six prognostic lncRNAs.

The 414 DLBCL patients from Lenz’s study^10^ (referred to as “Lenz dataset”) were divided randomly into a discovery series (n = 207) and an internal testing series (207) (see Supplementary Table S1). To identify prognosis-related lncRNAs, we first used univariate Cox proportional hazards regression model to evaluate the associations between the expression level of lncRNAs and overall survival (OS), and found that six lncRNAs were significantly associated with OS in the discovery series (adjusted p < 0.05 after Benjamini-Hochberg multiple testing correction, Table 1). To further investigate the expression pattern of these six lncRNAs, we performed an unsupervised hierarchical clustering for 207 DLBCL patients based on expression levels of these six lncRNAs in the discovery series. As shown in Fig. 1A, the resulting dendrogram showed two distinct patient clusters, which were highly correlated with patients’ survival status (p = 7.46e-03, Chi-square test). As previously described^11^,^12^, a panel of six-lncRNA signature was developed as a linear combination of the expression levels of these six lncRNAs and the estimated regression coefficients in the multivariate Cox regression analysis as the weight as follows: Risk Score = (SACS-AS1*0.4452) + (MME-AS1*−0.3143) + (CSMD2-AS1*−1.0086) + (RP11-360F5.1*−0.0827) + (RP11-25K19.1*−0.0181) + (CTC-467M3.1*−0.9599). We were able to calculate a lncRNA expression-based risk score (referred to as “LncRS”) for each patient in the discovery series and classified them into high-risk group (n = 104) or low-risk group (n = 103) using the median risk score of −1.2393 as the cutoff point. Patients were assigned to the high-risk group if their LncRSs were greater than or equal to the cutoff point, whereas low-risk group was composed of patients with LncRSs that were less than the cutoff point. As a result, patients in the high-risk group exhibited poor OS compared with those in the low-risk group (median OS 3.82 years vs. NA years, log rank p < 0.001) (Fig. 1B). There are 104 patients in the high-risk group and 103 patients in the low-risk group. The prognosis of patients in the discovery series showed 76 of dead (37%) and 131 of alive (63%). We performed odds ratio test to quantify the association between the real survival status of patients and the predicted risk group by the six-lncRNA signature. The odd ratio of the discovery series is 4.38 (95% CI = 2.37 to 8.11; p < 0.0001), suggesting that the high-risk group was more likely to have higher mortality than the low-risk group (53% vs. 20% for the discovery series) (see Supplementary Table S2).

The overall five- and ten-year relative survival rate of the high-risk group were 42.4% and 39.6%, respectively, whereas the corresponding rates in the low-risk group were 79.1% and 61.2%, respectively. Moreover, we found that the LncRS was significantly associated with OS (HR = 2.31, 95% CI = 1.8 to 2.965, p < 0.001) in a univariate analysis. Fig. 1C,D showed the LncRS distribution and expression pattern of six prognostic lncRNAs in patients of the discovery series (ranked according to increasing LncRS). As expected, five protective lncRNAs (*MME-AS1*, *CSMD2-AS1*, *RP11-360F5.1*, *RP11-25K19.1* and *CTC-467M3.1*) tended to be expressed in patients with low LncRS, whereas one risky lncRNAs (*SACS-AS1*) was up-regulated in patients with high LncRS.

### Prognostic value of the six-lncRNA signature for survival prediction in the internal testing series and entire Lenz dataset

**Figure 2:**
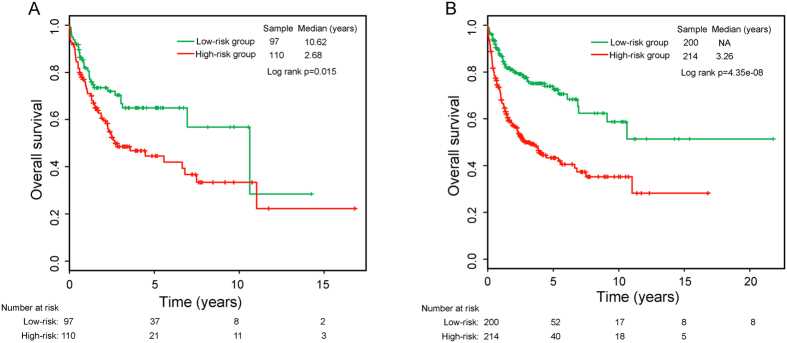
Survival prediction of the six-lncRNA signature in the testing series and entire Lenz dataset. Kaplan–Meier survival curves of overall survival between high-risk and low-risk patients in the testing series (**A**) and entire Lenz dataset (**B**).

The prognostic value of the six-lncRNA signature for survival prediction was evaluated using the internal testing series and entire Lenz dataset. With the same six-lncRNA signature score model and cutoff point derived from the discovery series, 207 patients of the internal testing series were divided into high-risk group (n = 110) and low-risk group (n = 97). As in the discovery series, OS in the high-risk group was significantly worse than that in the low-risk group (median OS 2.68 years vs. 10.62 years, log rank p = 0.015), and the proportions of OS in the high-risk group and low-risk group are 44.6% and 64.9% at five years, and 33.4% and 56.8% at ten years, respectively (Fig. 2A). The odd ratio of the internal testing series is 2.38 (95% CI = 1.35 to 4.19, p = 0.0028).

Similar results were observed in the entire Lenz dataset (i.e. combined discovery and internal testing series), which were comprised of 214 high-risk patients with median OS of 3.26 years and 200 low-risk patients with not reached median OS (log-rank p < 0.001) (Fig. 2B). The odd ratio of the entire Lenz dataset is 3.24 (95% CI = 2.14 to 4.90, p < 0.0001). In the univariate analysis, significant associations between the LncRS and OS also were found both in the testing series (HR = 1.362, 95% CI = 1.108 to 1.673, p = 0.003) and entire Lenz dataset (HR = 1.737, 95% CI = 1.484 to 2.034, p < 0.001). The LncRS distribution and expression pattern of six prognostic lncRNAs in patients of the testing series and entire Lenz dataset were shown in Supplementary Fig. S1, which were consistent with findings from the discovery series.

### Confirmation of the six-lncRNA signature for survival prediction in additional independent dataset

**Figure 3:**
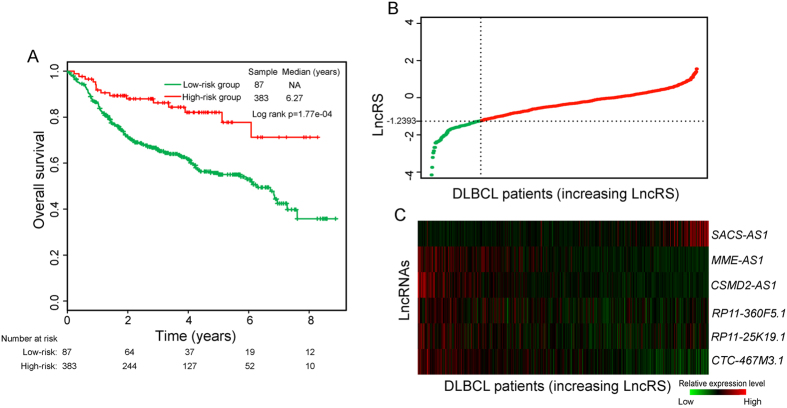
Independent confirmation of the six-lncRNA signature using an additional dataset of 470 patients. (**A**) Kaplan–Meier survival curves for patients in the Visco dataset. (**B**) The LncRS distribution of patients in the Visco dataset. (**C**) The expression heatmap of six prognostic lncRNAs in the 470 patients of Visco dataset.

We further evaluated the prognostic value of six-lncRNA signature for survival prediction in the additional independent patient dataset from Visco’s study^13^ (referred to as “Visco dataset”). The same six-lncRNA signature score model obtained from the discovery series was used to calculate the LncRS for each of 470 patients from the Visco dataset. With the same cutoff point derived from the discovery series, 470 patients of the Visco dataset were divided into the high-risk group (n = 383) and low-risk group (n = 87). As shown in Fig. 3A, patients with high-risk LncRS had significantly shorter survival than those belonging to low-risk group (median OS 6.27 years vs. NA years, log rank p < 0.001). The odd ratio of the Visco dataset is 3.26 (95% CI = 1.80 to 5.90, p = 0.0001). At four years and six years, the respective absolute differences in OS between the groups with high-risk and low-risk LncRS were 20.4% (61.7% vs. 82.1%) and 24.7% (53% vs. 77.7%), respectively. In univariate analysis of the Visco dataset, the LncRS were significantly correlated with OS (HR = 1.601, 95% CI = 1.315 to 1.95, p < 0.001) (Table 2). The distribution of LncRS and lncRNAs expression of patients in the Visco dataset is shown in Fig. 3B,C. Similar to the above findings, five protective lncRNAs were over-expressed and one risky lncRNA was down-regulated in the low-risk patients compared to the high-risk patients.

### Further confirmation of prognostic lncRNAs using additional independent dataset with a different array platform

**Figure 4:**
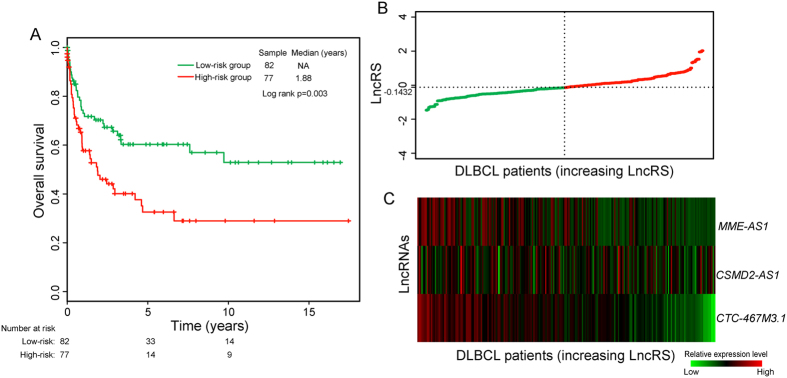
Independent cross-platform confirmation of the six-lncRNA signature. (**A**) Kaplan–Meier survival curves for patients in the Hummel dataset. (**B**) The LncRS distribution of patients in the Hummel dataset. (**C**) The expression heatmap of six prognostic lncRNAs in 159 patients of Hummel dataset.

To further examine the predictive power and robustness of six prognostic lncRNAs, we performed a cross-platform test analysis of prognostic lncRNAs identified in the discovery series using another existing DLBCL patient dataset measured with Affymetrix HG-U133A array from Hummel’s study^14^ (referred to as “Hummel dataset”). We re-annotated the probes of Affymetrix HG-U133A array to lncRNAs as described in the Materials and Method, and found that only 3 lncRNAs from the six-lncRNA signature were covered on the Affymetrix HG-U133A array. Therefore, the predictive power of these three prognostic lncRNAs (*MME-AS1*, *CSMD2-AS1* and *CTC-467M3.1*) was analyzed using the completely independent Hummel dataset of 159 DLBCL patients. The risk score of each patient in Hummel dataset was calculated based on the expression levels of these three lncRNAs according to the risk score model derived from the discovery series without re-estimating parameters. The median risk score obtained from Hummel dataset classified 159 DLBCL patients into the high-risk group (n = 77) and the low-risk group (n = 82). The Kaplan-Meier curves based on these three lncRNAs were markedly different (log rank p = 0.003), showing OS in 32.6% and 60.3% at five years, and 29% and 52.9% at ten years for patients with high-risk and low-risk LncRS, respectively (Fig. 4A). Furthermore, the univariate Cox regression analysis also showed that the risk scores were significantly associated with OS in DLBCL patients of the Hummel dataset (HR = 1.598, 95% CI = 1.098 to 2.327, p = 0.014). The results of LncRS distribution of patients and expression pattern of three prognostic lncRNAs also demonstrated their discriminatory power between patients with poor and good survival (Fig. 4B,C).

### Survival prediction by the six-lncRNA signature is independent of conventional clinical factors

**Figure 5:**
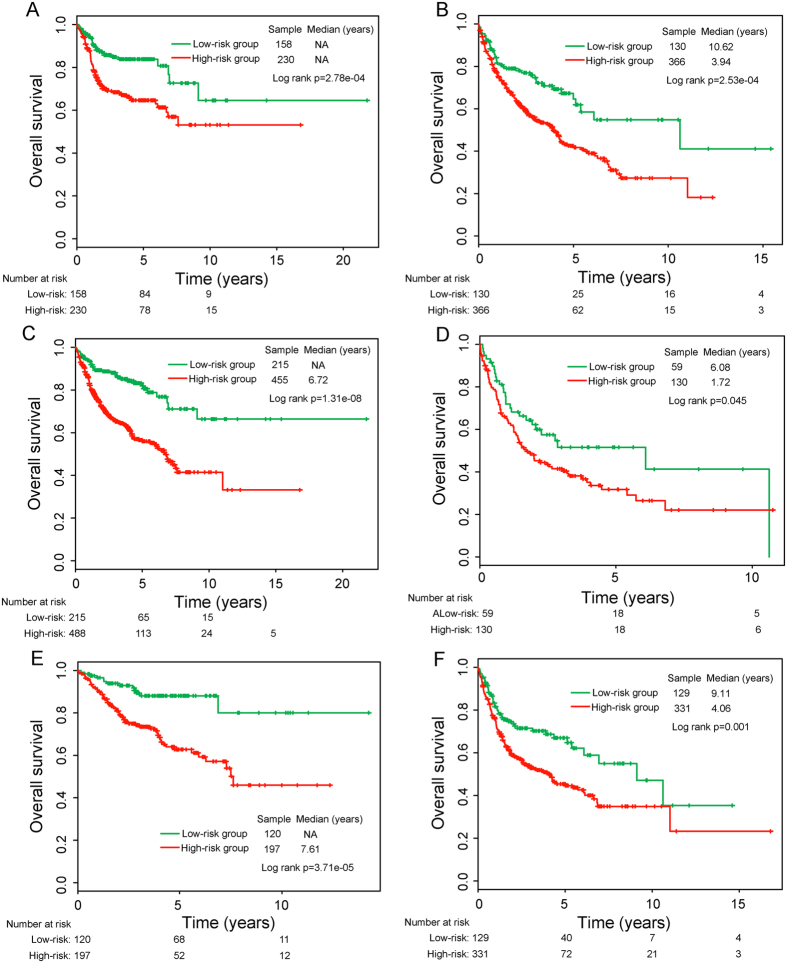
Survival prediction of the six-lncRNA signature in the stratification analysis of patients with available age, ECOG performance status and LDH level information. (**A**) Kaplan–Meier survival curves for younger patients with DLBCL. (**B**) Kaplan–Meier survival curves for elder patients with DLBCL. (**C**) Kaplan–Meier survival curves for DLBCL patients with ECOG performance status score <2. (**D**) Kaplan–Meier survival curves for DLBCL patients with ECOG performance status score of 2 or greater. (**E**) Kaplan–Meier survival curves for DLBCL patients with LDH < 1*normal. (**F**) Kaplan–Meier survival curves for DLBCL patients with LDH> = 1*normal.

To assess whether the prognostic values of the six-lncRNA signature is independent of conventional clinical factors of DLBCL patients, we performed the multivariate Cox regression analyses using OS as the dependent variable and LncRS and other conventional clinical factors as explanatory variables, and found that the six-lncRNA signature still maintained an independent correlation with OS both in the Lenz and Visco datasets after adjustment for conventional clinical factors, including age, gender, stage, number of extranodal sites, lactate dehydrogenase (LDH) level, Eastern cooperative Oncology Group (ECOG) performance status and subtype (Table 2). However, we found that age, LDH level and ECOG performance status were also significant in the multivariate analysis. Therefore, data stratification analysis was performed to examine whether the six-lncRNA signature could provide prognostic value within the same clinical factors. For this, all 884 patients (combining Lenz and Visco datasets) were stratified into younger patient group (< = 60) and elder patient group (>60) according to age. With the same six-lncRNA signature and risk score cutoff point derived from the discovery series, all 388 patients with age < = 60 were divided into the high-risk group (n = 230) with poor survival or the low-risk group (n = 158) with good survival (log-rank p < 0.001) (Fig. 5A). Similar prognostic value of the six-lncRNA signature was observed for elder patients who were classified into the high-risk group (n = 366) with median OS of 3.94 years and low-risk group (n = 130) with median OS of 10.62 years (log rank p < 0.001) (Fig. 5B). Further analysis found that the six-lncRNA signature was able to separate patients with ECOG performance status score <2 into the high-risk group (n = 455) with median OS of 6.72 years and low-risk group (n = 215) with not reached median OS (log rank p < 0.001, Fig. 5C). Similarity, among those patients with poor general health status (ECOG performance status score of 2 or greater), the six-lncRNA signature also could distinguish between patients with significantly different survival (median OS 1.72 years vs. 6.08 years, log rank p = 0.045, Fig. 5D) Another important clinical factor, LDH level, stratified all 777 patients with LDH information into two subgroups with LDH level lower than 1*normal or greater than 1*normal. Within each LDH stratum, survival analysis also demonstrated significant differences in OS between the high-risk group and low-risk group (median OS 7.61 years vs. NA years, log-rank p < 0.001 for 317 patients with LDH < 1*normal, and median OS 4.06 years vs. 9.11 years, log-rank p = 0.001 for 460 patients with LDH> = 1*normal) (Fig. 5E,F). Taken together, these results suggested that the predictive capacity of the six-lncRNA signature is independent of conventional clinical factors for survival prediction of DLBCL patients.

### Functional implication of the six-lncRNA signature

**Figure 6:**
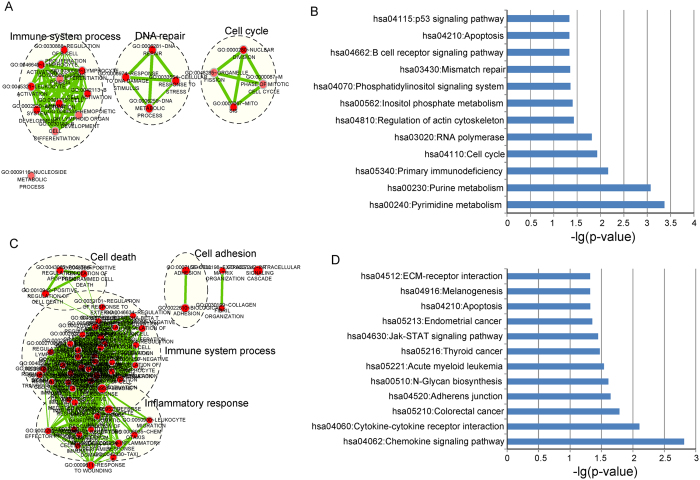
Functional enrichment analysis of protein-coding genes co-expressed with prognostic lncRNAs. (**A**) The functional enrichment map of GO terms enriched by positively correlated protein-coding genes. (**B**) Significantly enriched KEGG pathways of positively correlated protein-coding genes. (**C**) The functional enrichment map of GO terms enriched by negatively correlated protein-coding genes. (**D**) Significantly enriched KEGG pathways of negatively correlated protein-coding genes.

We further investigated the functional implication of the six-lncRNA signature in the development of DLBCL by Gene Ontology (GO) and Kyoto Encyclopedia of Genes and Genomes (KEGG) functional enrichment analysis for PCGs co-expressed with six prognostic lncRNAs. We first measured the co-expressed relationships between the expression level of prognostic lncRNAs and that of protein-coding genes (PCGs), and considered those PCGs whose co-expressed correlations with lncRNAs were ranked in the top 1% of all correlations as lncRNAs-related PCGs. The results of functional enrichment analysis demonstrated that PCGs positively correlated with prognostic lncRNAs were enriched in three GO functional clusters (including immune system process, DNA repair and cell cycle) (Fig. 6A) and 12 KEGG pathways (Fig. 6B). The PCGs negatively co-expressed with prognostic lncRNAs clustered most significantly in cell death, cell adhesion, immune system process, and inflammatory response for GO biological process enrichment analysis (Fig. 6C), and in 12 biological pathways for KEGG enrichment analysis (Fig. 6D). Most of enriched functional catalogues have been found to be closely associated with the incidence and development of DLBCL^15^, which emphasized an implication of the six-lncRNA signature in DLBCL through exerting their regulatory roles on PCGs involved in these known DLBCL-related biological processes and pathways.

## Discussion

Recent advances in the transcriptomic analysis have demonstrated the pathological and molecular heterogeneity of DLBCL^16^. Distinctive molecular heterogeneity is closely associated with a wide range of clinical characteristics and outcome. For almost two decades, the International Prognostic Index (IPI) system has been widely used to guide risk stratification and predict the outcome of DLBCL patients^17^. The IPI takes into account a series of clinical criteria, including age, stage, LDH level, ECOG performance status and number of extranodal sites^18^. However, the fact that the IPI system does not account for factors underlying the molecular heterogeneity of DLBCL patients, and considerable differences in survival outcome have been observed even among patients with the same or similar IPI variables, leading to increasing attention for identifying additional molecular prognostic biomarkers^18^. Several expression-based prognostic models have been developed to meet this need at the mRNA level^19^,^20^,^21^. Recent studies have revealed the contribution of lncRNAs to cancer development, implying their potential as novel biomarkers in cancer diagnosis^22^,^23^,^24^,^25^,^26^,^27^,^28^,^29^.

In this study, we investigated the prognostic value of lncRNAs by analyzing lncRNAs expression profiles and clinical characteristics in a large cohort of 1043 DLBCL patients. By using the sample splitting method and Cox regression analysis, we identified six prognostic lncRNAs that were significantly associated with OS of DLBCL patients. Based on these six prognostic lncRNAs, we constructed a six-lncRNA signature which was able to classify DLBCL patients into the high-risk and low-risk groups with significantly different survival outcome. The predictive value of the six-lncRNA signature was successfully validated in a completely independent dataset of 470 DLBCL patients. Furthermore, we used another independent dataset with different array platform (HG-U133A array) to validate our findings. Even though only three out of six prognostic lncRNAs were covered in this array, expression levels of these three lncRNAs were again shown to have close association with OS of patients. These results with independent DLBCL patient datasets demonstrated the robustness and good reproducibility of the six-lncRNA signature in DLBCL. Further analysis revealed that the six-lncRNA signature is independent of conventional clinical factors, including age, gender, stage, number of extranodal sites, LDH level, ECOG performance status and subtype. Notably, the six-lncRNA signature was shown capable of predicting survival for patients with the same or similar IPI variables, suggesting that the six-lncRNA signature could provide additional prognostic information at the molecular level beyond the conventional IPI system.

To date, research has only just begun into the biological function of lncRNAs and only a handful of lncRNAs were functionally well-characterized. Recent study found that lncRNA *CTC-467M3.1*, one of the six prognostic lncRNAs, is involved in the *cis*-regulation of transcription factor MEF2C^29^ which is required for B cell proliferation and survival^30^. Increasing evidence has suggested that lncRNAs were involved in diverse biological processes by negatively or positively regulating gene expression at both the posttranscriptional and transcriptional levels^31^,^32^. Therefore, it is feasible to infer biological roles of lncRNAs by functional views of PCGs that are co-expressed with lncRNAs^33^,^34^,^35^. So we investigated the co-expression patterns of mRNAs and lncRNAs and performed functional enrichment analysis for co-expressed PCGs to predict biological function of prognostic lncRNAs in this signature. We found that PCGs whose expression value positively or negatively correlated with the six prognostic lncRNAs were enriched in three functional groups of known cancer-related pathways, immune system-related biological processes and signaling molecules interaction that are closely linked with the incidence and progression of DLBCL^15^. Thus, it is a plausible inference that the six-lncRNA signature may be involved with DLBCL through exerting their regulatory roles on PCGs in these known DLBCL-related biological processes and pathways.

In conclusion, our study investigated the prognostic potential of lncRNAs in DLBCL, and identified a potential panel of six-lncRNA signature as a composite biomarker for risk stratification of DLBCL patients at diagnosis. Moreover, the six-lncRNA signature was able to effectively predict the survival outcome of DLBCL patients with similar IPI variables. To our knowledge, this is the first report on efforts to identify lncRNA signature that predicts survival outcome of patients with DLBCL. With further confirmation, the six-lncRNA signature not only provides additional prognostic value beyond the conventional IPI system to identify high-risk patients who will benefit from more effective therapy, but also can improve our understanding about the molecular heterogeneity of DLBCL from the viewpoint of non-coding RNA. However, it should be noted that a large portion of known lncRNAs were missing in our study due to the intrinsic limitation of the microarray technique and probe repurposing method. Therefore, more research is needed to uncover novel diagnostic or prognostic lncRNAs candidates in DLBCL.

## Materials and Methods

### DLBCL patient datasets

Genome-wide gene expression profiles data generated from the Affymetrix platform (Affymetrix HG-U133 Plus 2.0 array and HG-U133A array) and corresponding clinical information of DLBCL patients were retrieved from the GEO database. After removing patients without available clinical information, a total of 1043 DLBCL patients were enrolled in this study, including 414 patients from Lenz’s study (the GEO accession number is GSE10846, Affymetrix HG-U133 Plus 2.0 array) (http://www.ncbi.nlm.nih.gov/geo/query/acc.cgi?acc =  GSE10846)^10^, 470 patients from Visco’s study (the GEO accession number is GSE31312, Affymetrix HG-U133 Plus 2.0 array)(http://www.ncbi.nlm.nih.gov/geo/query/acc.cgi?acc =  GSE31312)^13^ and 159 patients from Hummel’s study (the GEO accession number is GSE4475, Affymetrix HG-U133A array) (http://www.ncbi.nlm.nih.gov/geo/query/acc.cgi?acc =  GSE4475)^14^. Detailed clinical information of DLBCL patients used in this study was shown in Supplementary Table S1.

### Acquisition of lncRNA expression profiles of DLBCL patients

The raw array data (.CEL files) of 1043 DLBCL patients on the Affymetrix HG-U133 Plus 2.0 array or HG-U133A array were downloaded from the GEO database and were uniformly pre-processed using the Robust Multichip Average (RMA) algorithm for background correction, quantile normalization and log2-transformation^36^. To account for the heterogeneity in systematic measurement among multiple microarray datasets, each dataset was standardized independently by the Z-score transformation to scale expression intensities of each probe into having a mean of 0 and a standard deviation of 1.

The probe sequences of Affymetrix HG-U133 Plus 2.0 and HG-U133A arrays were retrieved from the Affymetrix website (http://www.affymetrix.com). We re-mapped those probes into the human genome (GRCh38) to obtain the chromosomal position of the probes using SeqMap tool^37^. Then the lncRNAs-specific probes were obtained by matching the chromosomal position of probes to the chromosomal position of lncRNAs based on the annotation from the GENCODE project (http://www.gencodegenes.org, release 22) as previously described^22^,^23^,^38^. The expression levels of lncRNAs were then obtained from the normalized intensity of lncRNAs-specific probes. Finally, 2330 lncRNAs for Affymetrix HG-U133 Plus 2.0 and 663 lncRNAs for Affymetrix HG-U133A were obtained for subsequent analysis.

### Statistical analysis

The association between the expression level of each lncRNA and OS of DLBCL patients was evaluated using the univariate Cox regression analysis and Benjamini-Hochberg multiple testing correction. LncRNAs were considered as prognostic lncRNAs if their adjusted p-value were less than 0.05. Then a six-lncRNA expression signature was constructed using a linear combination of the expression levels of these six lncRNAs and the estimated regression coefficients in the multivariate Cox regression analysis as previously described^11^,^12^. With this six-lncRNA signature, patients in each dataset were classified into high-risk group and low-risk group by using the median LncRS of the discovery series as the cutoff point. Kaplan-Meier survival curves and log-rank test were used to assess the difference in OS between the two groups with high-risk and low-risk LncRS. Univariate and multivariate analyses with Cox proportional hazards regression for OS were performed on the individual conventional clinical variables with and without the six-lncRNA signature in each dataset. Hazard ratios (HR) and 95% confidence intervals (CI) were calculated. All statistical tests were two-sided and performed with R software.

### Functional enrichment analysis

The Pearson correlation coefficients between the expression level of each lncRNAs and that of each PCG were calculated. The PCGs positively or negatively correlated with prognostic lncRNAs (ranked top 1%) were considered as lncRNAs-related PCGs. Functional enrichment analysis of lncRNAs-related PCGs for GO terms and KEGG pathway was performed using DAVID Bioinformatics Tool (https://david.ncifcrf.gov/, version 6.7)^39^. GO functional clusters limited to “Biological Process”(GOTERM-BP-FAT) with an enrichment score of >1.5 and KEGG pathway Functional Annotation with p-value of <0.05 using the whole human genome as background were considered as potential functional roles of prognostic RNAs. Significant GO terms with similar function were organized into an interaction network and visualized using the Enrichment Map^40^.

## Additional Information

**How to cite this article**: Sun, J. *et al.* A potential panel of six-long non-coding RNA signature to improve survival prediction of diffuse large-B-cell lymphoma. *Sci. Rep.* **6**, 27842; doi: 10.1038/srep27842 (2016).

## Supplementary Material

Supplementary Information

## Figures and Tables

**Table 1:** Overall information of six prognostic lncRNAs associated with survival outcome of DLBCL patients.

Ensembl ID	Gene symbol	Chromosome	P value^a^	Hazard ratio^a^	Coefficient^a^
ENSG00000229558	SACS-AS1	Chr 13: 23,418,971–23,428,869 (+)	1.9e-06	1.81	0.593
ENSG00000240666	MME-AS1	Chr 3: 155,158,370–155,183,285 (−)	5.8e-06	0.581	−0.542
ENSG00000231163	CSMD2-AS1	Chr 1: 33,868,953–33,885,458 (+)	7.4e-06	0.208	−1.571
ENSG00000249207	RP11-360F5.1	Chr 4: 39,112,677–39,126,818 (−)	9.12e-06	0.243	−1.415
ENSG00000167912	RP11-25K19.1	Chr 8: 59,119,040–59,121,346 (+)	4.75e-05	0.528	−0.639
ENSG00000245864	CTC-467M3.1	Chr 5: 88,676,218–88,722,831 (+)	5.32e-05	0.281	−1.268

^a^Derived from the univariate Cox regression analysis in 207 patients of the discovery series.

**Table 2:** Univariate and multivariate Cox regression analysis of overall survival in each dataset.

Variables	Univariate analysis			Multivariate analysis		
	HR	95% CI of HR	P value	HR	95% CI of HR	P value
**Lenz dataset (n = 414)**						
Six-lncRNA risk score	1.737	1.484–2.034	6.55e-12	1.585	1.203–2.088	0.001
Age (≤ 60 vs. >60)	0.452	0.326–0.628	2.25e-06	0.478	0.309–0.739	9.02e-04
Gender (Female vs. Male)	1.021	0.744–1.402	0.897	1.116	0.756–1.648	0.582
Stage (I/II vs. III/IV)	0.545	0.394–0.754	2.42e-04	0.909	0.597–1.385	0.657
No. of extranodal sites (<2 vs. ≥2)	0.518	0.308–0.873	0.014	1.05	0.478–2.305	0.904
LDH	1.137	1.095–1.181	2.82e-11	1.156	1.095–1.22	1.76e-07
ECOG (<2 vs. ≥2)	0.353	0.255–0.488	3.03e-10	0.457	0.299–0.697	2.81e-04
Subtype (GCB vs. ABC)	0.364	0.257–0.514	1.09e-08	0.872	0.481–1.579	0.651
**Visco dataset (n = 470)**						
Six-lncRNA risk score	1.601	1.315–1.95	2.85e-06	1.467	1.122–1.917	0.005
Age (≤ 60 vs. >60)	0.541	0.391–0.749	2.1e-04	0.57	0.4–0.813	0.002
Gender (Female vs. Male)	0.966	0.712–1.31	0.824	0.869	0.627–1.206	0.402
Stage (I/II vs. III/IV)	2.337	1.688–3.238	3.25e-07	1.743	1.178–2.579	0.005
No. of extranodal sites (<2 vs. ≥2)	0.454	0.329–0.626	1.49e-06	0.622	0.432–0.895	0.011
LDH	2.129	1.453–3.121	1.07e-04	1.507	1.01–2.25	0.045
ECOG (<2 vs. ≥2)	0.491	0.352–0.685	2.76e-05	0.631	0.43–0.925	0.018
Subtype						
GCB vs. ABC	0.583	0.423–0.802	9.29e-04	0.967	0.627–1.492	0.88
UC vs. ABC	0.766	0.449–1.306	0.327	0.856	0.484–1.517	0.595
**All samples (n = 884)**						
Six-lncRNA risk score	1.516	1.35–1.702	2.14e-12	1.374	1.158–1.631	2.73e-04
Age (≤ 60 vs. >60)	0.503	0.4–0.634	5.36e-09	0.536	0.407–0.704	7.73e-06
Gender (Female vs. Male)	0.991	0.795–1.23	0.934	0.932	0.727-1.196	0.581
Stage (I/II vs. III/IV)	0.482	0.383–0.607	4.62e-10	0.662	0.499–0.878	0.004
No. of extranodal sites (<2 vs. ≥2)	0.519	0.399–0.676	1.08e–06	0.737	0.539–1.007	0.056
LDH	1.154	1.117–1.193	2.0e-16	1.156	1.111–1.204	1.51e-12
ECOG (<2 vs. ≥2)	0.411	0.326–0.518	4.6e-14	0.511	0.386–0.675	2.29e-06
Subtype						
GCB vs. ABC	0.466	0.369–0.589	1.61e-10	0.802	0.575–1.12	0.195
UC vs. ABC	0.629	0.377–1.048	0.075	0.713	0.414–1.226	0.221

Abbreviations: HR, hazard ratio; CI, confidence interval.
