# Supplementary material for: A potential panel of six-long non-coding RNA signature to improve survival prediction of diffuse large-B-cell lymphoma
Source: Sci Rep. 2016 Jun 13;6:27842. doi: 10.1038/srep27842 (PMC4904406; doi:10.1038/srep27842)
Supplement: Supplementary Information [file srep27842-s1.doc]

**A potential panel of six-long non-coding RNA signature to improve survival prediction of** **diffuse large-B-cell lymphoma**

Jie Sun ^1^, ^§, *^, Liang Cheng^1, §^, Hongbo Shi^1, §^, Zhaoyue Zhang^1^, Hengqiang Zhao^1^, Zhenzhen Wang^1^, Meng Zhou^1, *^

^1^College of Bioinformatics Science and Technology, Harbin Medical University, Harbin 150081, PR China

***Corresponding author**

Jie Sun, suncarajie@hotmail.com

College of Bioinformatics Science and Technology, Harbin Medical University, Harbin 150081, PR China

Meng Zhou, biofomeng@hotmail.com

College of Bioinformatics Science and Technology, Harbin Medical University, Harbin 150081, PR China

**^§^These authors contributed equally to this work**

**Supplementary Table S1.** Clinical characteristics of DLBCL patients in each dataset

| Characteristics | Discovery series | Testing series | Lenz dataset | Visco dataset | Hummel dataset |
| --- | --- | --- | --- | --- | --- |
| **No. of patients** | 207 | 207 | 414 | 470 | 221 |
| **Age, year** |  |  |  |  |  |
| >60 | 109 | 117 | 226 | 270 | 112 |
| ≤60 | 98 | 90 | 188 | 200 | 107 |
| NA |  |  |  |  | 2 |
| **Gender** |  |  |  |  |  |
| Female | 79 | 93 | 172 | 199 | 91 |
| Male | 117 | 107 | 224 | 271 | 124 |
| Unknown | 11 | 7 | 18 | 0 | 6 |
| **Stage** |  |  |  |  |  |
| I/II | 105 | 83 | 188 | 220 | 65 |
| III/IV | 97 | 121 | 218 | 250 | 78 |
| Unknown | 5 | 3 | 8 |  | 78 |
| **No. of extranodal sites** |  |  |  |  |  |
| <2 | 176 | 177 | 353 | 366 |  |
| ≥2 | 15 | 15 | 30 | 104 |  |
| Unknown | 16 | 15 | 31 | 0 |  |
| **LDH** |  |  |  |  |  |
| median(range) | 1.035(0, 30.76) | 1.0(1, 14.84) | 1.01(0, 30.76) |  |  |
| 0 |  |  |  | 148 |  |
| 1 |  |  |  | 278 |  |
| Unknown | 31 | 32 | 63 | 44 |  |
| **ECOG** |  |  |  |  |  |
| <2 | 149 | 147 | 296 | 374 |  |
| ≥2 | 42 | 51 | 93 | 96 |  |
| Unknown | 16 | 9 | 25 | 0 |  |
| **Subtype** |  |  |  |  |  |
| GCB | 92 | 91 | 183 | 227 | 120 |
| ABC | 84 | 83 | 167 | 199 | 58 |
| Unclassified | 31 | 33 | 64 | 44 | 43 |
| **Survival Status** |  |  |  |  |  |
| Dead | 76 | 89 | 165 | 300 | 75 |
| Alive | 131 | 118 | 249 | 170 | 84 |

**Supplementary Table S2**. 4 Square table and odds ratio in each dataset

|  | Discovery series (n=207) | | |
| --- | --- | --- | --- |
|  | High-risk group | Low-risk group | Odd ratio test |
| Dead case | 55 | 21 | OR=4.38; P < 0.0001;  95% CI, [2.37, 8.11]; |
| Alive case | 49 | 82 |
|  | Internal testing series (n=207) | | |
|  | High-risk group | Low-risk group | Odd ratio test |
| Dead case | 58 | 31 | OR=2.38; P=0.0028  95% CI, [1.35, 4.19]; |
| Alive case | 52 | 66 |
|  | Lenz dataset (414) | | |
|  | High-risk group | Low-risk group | Odd ratio test |
| Dead case | 113 | 52 | OR=3.18; P<0.0001  95% CI, [2.10, 4.82]; |
| Alive case | 101 | 148 |
|  | Visco dataset (470) | | |
|  | High-risk group | Low-risk group | Odd ratio test |
| Dead case | 72 | 15 | OR=3.26; P=0.0001  95% CI, [1.80, 5.90]; |
| Alive case | 228 | 155 |


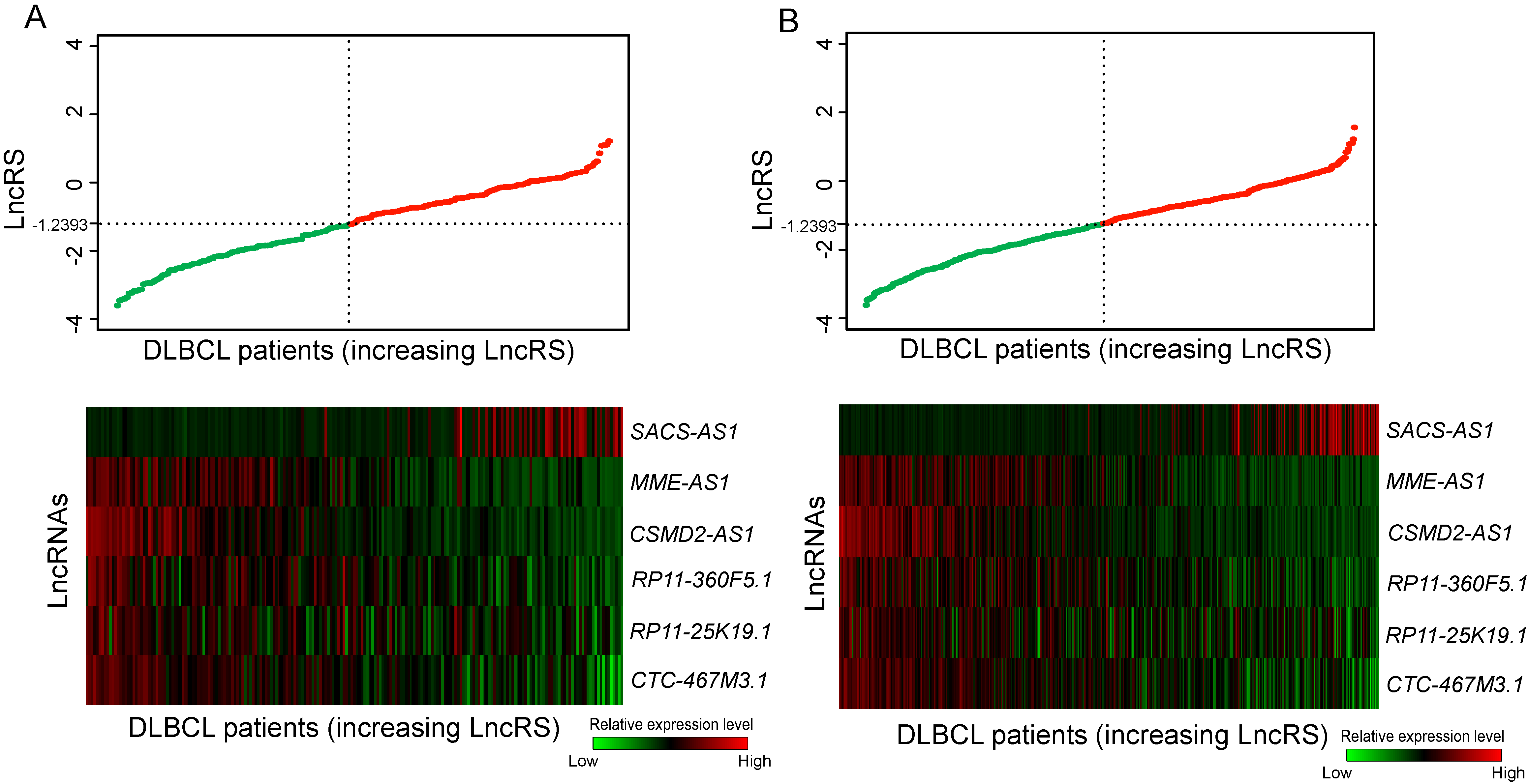


**Supplementary Figure S1.** The LncRS distribution of patients and the expression heatmap of six prognostic lncRNAs in the testing series (A) and entire Lenz dataset (B).
